# The efficacy and safety of microwave ablation in managing osteoid osteoma: a systematic review

**DOI:** 10.1080/23320885.2025.2503195

**Published:** 2025-05-10

**Authors:** Berun A. Abdalla, Hiwa O. Abdullah, Hawkar A. Nasralla, Rebaz M. Ali, Sami S. Omar, Abdullah K. Ghafour, Saywan K. Asaad, Soran S. Raoof, Soran H. Tahir, Rezheen J. Rashid, Ayoob A. Mohammed, Nasren Sharef Sabr, Ali H. Hasan, Lawen Jamal Mustafa, Ameer M. Salih, Fahmi H. Kakamad

**Affiliations:** a Scientific Affairs Department, Smart Health Tower, Madam Mitterrand Street, Sulaymaniyah, Iraq; b Kscien Organization for Scientific Research (Middle East Office), Hamid Street, Azadi mall, Sulaymaniyah, Iraq; c Medicine Department, Hiwa Cancer Hospital, Sulaymaniyah, Iraq; d Oncology Department, Rizgary Teaching Hospital, Erbil, Iraq; e College of Medicine, University of Sulaimani, Sulaymaniyah, Iraq; f Rheumatology Department, Ministry of Health, Sulaymaniyah, Iraq

**Keywords:** Microwave ablation, radiofrequency ablation, osteoid osteoma, minimal invasive, clinical efficacy

## Abstract

Introduction: Microwave ablation (MWA) utilizes electromagnetic methods to destroy tumors, employing devices operating at 900 MHz or above frequencies. MWA has emerged as a recent alternative for treating osteoid osteoma (OO), providing similar accessibility, safety, and technical effectiveness as radiofrequency ablation. This systematic review aims to evaluate the safety and efficacy of MWA in treating OO. Methods: A systematic review of published studies on the use of MWA in managing OO was conducted. Studies were excluded if they were 1) not written in English, 2) case reports, 3) lacked adequate peer review, or 4) consisted solely of abstracts. Before full-text assessment, titles, and abstracts were screened. Extracted data included author, year, study design, patient count, age, gender, OO site, ablation power (watts), duration, complications, outcome, and recurrence. The data were analyzed and presented as means, frequencies, and percentages. Results: Eight studies, including 143 cases, met the inclusion criteria; among these cases, 59.44% were male, and the mean age was 19.03 ± 7.09. Most of the OOs were in the femur, 54.54%. MWA at 60 W was the prevailing power setting, utilized in 37 cases (25.87%), with an ablation time of 90 s for 95 cases (66.43%). Clinical success was achieved in 137 (95.80%) cases, with recurrence observed in 4 cases (2.80%). However, 16 minor and major complications were observed despite the overall success. Conclusion: Percutaneous MWA may represent an efficient choice for the minimally invasive management of OO, demonstrating a minimal risk of complications and recurrence.

## 1. Introduction

Tumor ablation involves the direct application of chemical or thermal treatments to a tumor to either eradicate it or cause substantial destruction. This principle has been established for over a century [1]. Several ablation techniques, including cryoablation, ethanol ablation, laser ablation, and radiofrequency ablation (RFA), have been used. A recent advancement in this field is the use of microwave energy for tumor ablation [2].

Microwave ablation (MWA) involves utilizing electromagnetic techniques to induce tumor destruction using devices operating at frequencies of 900 MHz or higher [2]. It was initially used in the liver for hepatocellular carcinoma. However, in treating osteoid osteoma (OO), it has emerged as a recent therapeutic alternative that offers comparable accessibility, safety, and technical efficacy to RFA [3].

OOs are benign growths that frequently cause discomfort. Benign tumors are localized, non-cancerous growths that do not invade surrounding tissues. The OO was first described by Jaffe in 1935 [4]. They are ranked as the third most prevalent non-cancerous bone tumor; these typically small (less than 1.5 cm) solitary growths are known for causing intense nighttime pain, which can be alleviated by nonsteroidal anti-inflammatory drugs (NSAIDs) [5,6]. The tumor consists of three concentric regions: the innermost nidus, a neoplastic meshwork comprising osteoblasts, dilated vessels, and osteoid; a middle fibrovascular rim; and outermost reactive sclerosis. The primary cause of pain stems from the release of prostaglandins by the nidus [7].

Typically, OOs represent about 13.5% of benign bone tumors. They predominantly affect males in their third decade of life, with a male-to-female ratio of 4:1. They are commonly found in the long bones of the lower extremities; other frequent locations include the hands, feet, and spine [8].

For many years, surgery was the conventional treatment for such lesions. The introduction of RFA was first reported in 1989, with initial results published in 1992. Today, this minimally invasive procedure is widely accessible, safe, and efficient [9]. Using the same technique and coaxial introduction systems, microwave antennas can be inserted instead of radiofrequency needles [10].

This systematic review aims to evaluate the safety and effectiveness of MWA in treating OO.

## 2. Method

### 2.1. Study design

The current systematic review followed the protocols outlined in the Preferred Reporting Items for Systematic Reviews and Meta-Analyses (PRISMA) guidelines.

### 2.2. Data sources and search strategy

A comprehensive search was performed on ‘Google Scholar’ and ‘PubMed’ to identify all pertinent English-language studies regarding the application of MWA in managing OO. The keywords used were ‘microwave ablation OR therapy osteoid osteoma’.

### 2.3. Eligibility criteria

All studies on utilizing MWA in OOs were included, except for those that 1) were not in English, 2) constituted case reports, 3) lacked appropriate peer review, or 4) were solely abstracts. All included studies underwent eligibility verification [11].

### 2.4. Study selection and data extraction

Before a thorough full-text assessment for eligibility, the titles and abstracts of the included studies were initially screened. Various data were extracted from the included studies, such as author, year of publication, study design, number of patients, age, gender, site of the OOs, the power setting of the ablation in watts, duration of the ablation, complications, outcome, and recurrence.

### 2.5. Statistical analyses

The gathered data underwent analysis using Statistical Package for the Social Sciences software version 25.0 and were subsequently depicted in mean, frequency, and percentages.

## 3. Results

**Figure 1.**
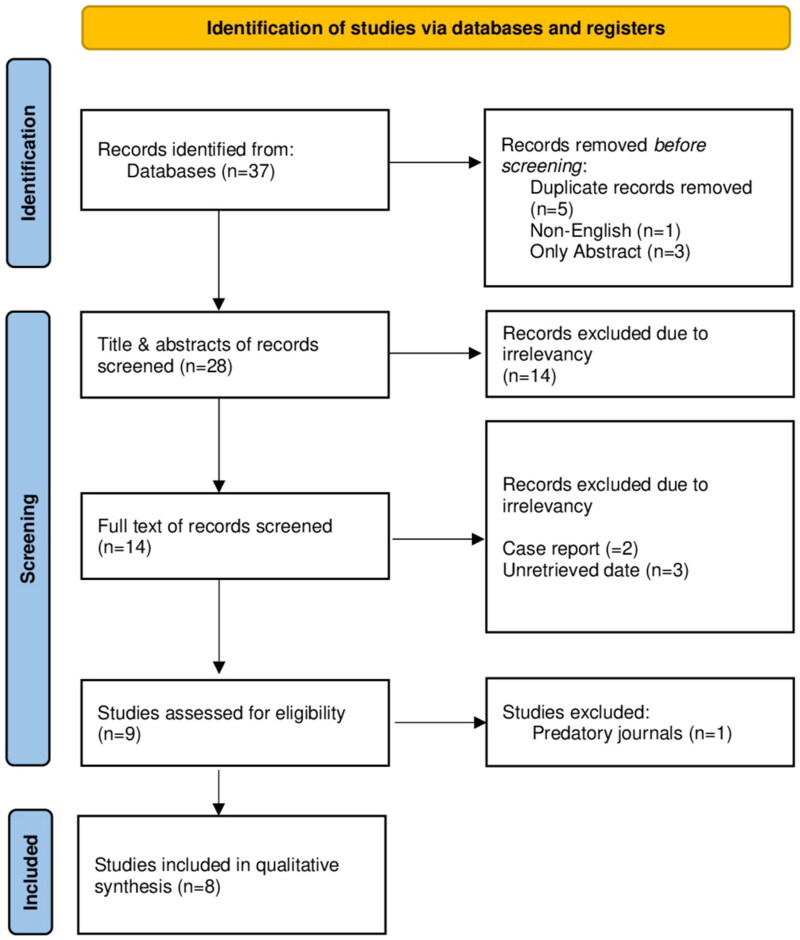
The PRISMA flow chart illustrates the process of study selection.

A total of 37 studies were identified from the sources, with nine excluded immediately due to duplication, non-English language, or being limited to abstracts. An initial screening of the remaining 28 studies based on titles and abstracts resulted in the exclusion of 14 studies deemed ineligible. Consequently, 14 studies underwent full-text review, of which five were excluded due to being case reports or lacking retrievable data. The remaining nine studies were assessed for eligibility, with one further excluded due to an inappropriate peer review process. Ultimately, eight studies [3–5,10, 13–16], encompassing 143 cases, met the inclusion criteria (Figure 1). Among these cases, 85 (59.44%) were male, while 58 (40.56%) were female. The mean age was 19.03 ± 7.09 years (Table 1). Most OOs were in the femur, accounting for 78 cases (54.54%). General anesthesia emerged as the preferred choice of analgesia, administered in 108 cases (75.52%). Fluoroscopy CT guidance was utilized in 80 cases (55.94%). MWA at 60 W was used in 37 cases (25.87%), with an ablation time of 90 s in 95 cases (66.43%). Clinical success was achieved in 137 cases (95.80%), while recurrence occurred in 4 cases (2.80%). Sixteen complications (11.19%) were observed, including 13 minor and three major complications. The most common minor complications were grade two skin burns and localized skin numbness, each affecting four patients (2.80%). For the major complications, grade three skin burns were reported in two patients (1.40%), and osteomyelitis occurred in one patient (0.70%) (Table 2).

**Table 1.** The raw data of the included studies..

Author, year ^(Reference)^	Study design	Case No.	Gender		Age (mean years)	Type of anesthesia					MWA power (Watt)	Time Sec. (mean)	Outcome		Complication		Recurrence	
			Male	Female		GA	SA	LA	SE	N/A			Effective	Non-E	yes	No	Yes	No
Parisot et al. 2022 [3]	cohort	28	18	10	19.5	21	1	6	0	0	60	90	26	2	3	25	2	26
Özbalci et al. 2022 [4]	cohort	23	10	13	16.22	23	0	0	0	0	30 to 50	90	23	0	3	20	0	23
Rinzle et al. 2018 [5]	cohort	24	15	9	13.3	24	0	0	0	0	30	90	24	0	4	20	0	24
Basile et al. 2014 [10]	case series	7	4	3	21.28	0	7	0	0	0	20	120	7	0	0	7	0	7
Kostrzewa et al., 2019 [13]	cohort	24	16	8	27.95	24	0	0	0	0	16	64.79	21	3	0	24	1	23
Prud’homme et al. 2016 [14]	case series	13	10	3	20.69	7	1	5	0	0	50 and 60	100	13	0	2	11	0	13
Reis et al. 2020 [15]	cohort	15	8	7	17.6	0	0	0	15	0	30 to 50	90	14	1	2	13	1	14
Yildirim et al. 2022 [16]	cohort	9	4	5	14.55	9	0	0	0	0	15	N/A	9	0	0	9	0	9

GA: general anesthesia; SA: spinal anesthesia; LA: local anesthesia; SE: sedation; N/A: not available; Non-E: non-effective.

**Table 2.** Summary outlining the fundamental characteristics of the studies that were included.

Variables	Frequency/percentage
Age (mean of means) ± SD	19.03 ± 7.09
Gender	
Female	58 (40.56%)
Male	85 (59.44%)
Site of osteoid osteoma	
Femur	78 (54.54%)
Tibia	38 (26.57%)
Talus	5 (3.49%)
Fibula	4 (2.80%)
Scapula	4 (2.80%)
Humerus	3 (2.10%)
Lesser trocanter	2 (1.40%)
Radius	2 (1.40%)
Acetabulum	1 (0.70%)
Calcaneus	1 (0.70%)
Navicular bone	1 (0.70%)
Fourth dorsal rib	1 (0.70%)
Right 4th metatarsal bone base	1 (0.70%)
N/A	2 (1.40%)
Type of anesthesia	
General anesthesia	108 (75.52%)
Spinal anesthesia	9 (6.29%)
Local anesthesia	11 (7.69%)
Only sedation	15 (10.48%)
Imaging guidance	
Fluoroscopy CT-guidance	80 (55.94%)
CT-scan	54 (37.76%)
C-arm fluoroscopy	9 (6.29%)
Power of microwave ablation	
Unspecified	38 (26.57%)
60 W	37 (25.87%)
16 W	24 (16.78%)
30 W	24 (16.78%)
15 W	9 (6.29%)
50 W	4 (2.80%)
20 W	7 (4.90%)
Ablation time	
90 s	95 (66.43%)
60 s	24 (16.78%)
120 s	11 (7.69%)
30 s	1 (0.70%)
45 s	1 (0.70%)
160 s	1 (0.70%)
250 s	1 (0.70%)
N/A	9 (6.29%)
Efficacy	
Effective	137 (95.80%)
Ineffective	6 (4.19%)
Complications*	
Minor complications	
Grade 2 skin burn	4 (2.80%)
Local skin numbness	4 (2.80%)
Superficial and partial radial nerve injury	2 (1.40%)
Bone marrow edema	1 (0.70%)
Infection at the access site	1 (0.70%)
Mild femoral neurapraxia	1 (0.70%)
Major complications	
Grade 3 skin burn	2 (1.40%)
Osteomyelitis	1 (0.70%)
No complications	129 (90.21%)
Recurrence	
No	139 (97.20%)
Yes	4 (2.80%)

W: watt; N/A: not available.

* Some of the patients had more than one complication.

## 4. Discussion

Pain represents the primary and consistent complaint associated with OOs, with its characteristics varying based on the tumor’s location. Typically, OO pain is localized, persistent, particularly prominent at night, and alleviated by aspirin or other NSAIDs [16]. Unfortunately, the tumor is not always detected promptly, often due to factors such as the mechanism of pain and the tumor’s positioning, leading to delays in diagnosis and treatment. Regardless of its location, OO-induced pain stems from two key factors: nerve endings within the cancer and the elevated levels of prostaglandins in the nidus. These trigger an inflammatory response that accounts for the responsiveness to NSAIDs [16].

In more than half of the instances, OOs are located at the core of the diaphysis of the femur or tibia. Less frequently, they appear in the axial skeleton and have even been documented in the cortical bone of the external auditory canal [12]. They are more prevalent in males than females, and while 75% arise between the ages of 5 and 25, they can manifest in the mature skeleton up to 70. [12]. In this study, 54.54% of the OOs were situated in the femur, followed by 26.57% in the tibia. The average age of the patients was 19.03 ± 7.09.

Computed tomography (CT) is typically the preferred imaging method for diagnosing OO [17]. However, radiation-free imaging modalities are necessary due to radiation dose considerations, especially in young patients. Several studies have demonstrated that high-resolution T1-weighted dynamic contrast-enhanced magnetic resonance imaging (DCE-MRI) can also effectively aid in diagnosing OO [17].

While surgery remains the conventional treatment for OO, it involves longer operating times, extended hospital stays, and higher costs. Moreover, the overall recovery period is significantly prolonged, accompanied by a notable incidence of morbidity and complications such as fracture, infection, and hematoma, with rates ranging from 20% to 45% [15]. Thermal ablation has emerged as the preferred initial treatment approach for OO, encompassing various modalities such as RFA, MWA, and laser ablation [7]. MWA is gaining traction as the treatment option for OO due to its favorable efficacy and safety profile. In MWA, percutaneous microwave antennae are positioned under CT guidance to administer high-energy alternating electromagnetic waves, producing frictional heat and achieving peak temperatures up to 150 °C [7].

MWA induces cell death via coagulative necrosis by raising intratumoral temperatures through the agitation of water molecules due to exposure to an electromagnetic field. This unique mechanism enables microwaves to penetrate deeper and heat tissues more effectively, leading to greater areas of tissue ablation as they are less hindered by impedance [9]. The key distinction between MWA and RFA lies in their heating mechanisms. MWA functions by exciting water molecules within the range of the microwave field, thereby heating the tissue. In contrast, RFA only penetrates a few millimeters into the surrounding tissue, with heat primarily spreading through it *via* conduction [18]. Common issues linked with RFA include the charring of the needle tip when power application decreases and ablation times extend, the creation of an uneven ablation volume, and the heat-sink effect [8]. A systematic literature review on RFA revealed an average ablation duration of 6.8 min for treating OO [19]. Although the literature describing ablation time in MWA for OOs is more limited, a range of 1–3 min has been reported [4]. Parisot et al. retrospectively analyzed 28 consecutive patients with a median age of 19.5 years who underwent treatment for 28 non-spinal osteoid osteomas using CT-guided MWA. The procedures utilized a median power of 60 watts and a median duration of 1 min and 30 s [3]. In the current study, a power setting of 60 W was the most frequently used for MWA, with an average ablation time of about 1.5 min. The majority of procedures (95.63%) had a duration of 90 s. Additionally, fluoroscopy CT guidance was employed in 55.94% of cases.

One major concern with MWA is the potential for uneven heat distribution, which can cause overheating and unintended thermal damage to surrounding healthy tissues. The complexity of MWA systems may lead to inconsistent outcomes. Furthermore, the risk of probe overheating remains, as excessive heat can damage tissues along the probe’s path [20]. Additionally, while the heat-sink effect is more commonly associated with RFA, it can also affect MWA by allowing blood flow from nearby vessels to dissipate heat from the target lesion, potentially resulting in inadequate tumor treatment. Understanding these risks is crucial for clinicians to optimize the benefits of MWA while minimizing complications. These factors underscore the importance of careful planning and technique adjustments during MWA procedures to ensure effective tumor destruction while protecting adjacent structures [20].

RFA has proven to be a highly effective and safe treatment for osteoid osteomas, particularly when performed under CT guidance. In a study of 116 patients, Sahin et al. reported a 100% technical success rate and a 98% efficacy rate, with most patients experiencing significant pain relief within 24 h of the procedure. The complication rate was low, with only seven minor complications, all managed conservatively [21]. Similarly, Çakar et al. found that RFA provided complete pain relief in all treated patients, with no significant complications during follow-up, highlighting its safety and efficacy as a minimally invasive alternative to surgery [22]. RFA has also demonstrated excellent results in recurrent cases, achieving a success rate of 96.6% after one or two sessions, further emphasizing its reliability [23]. Additionally, Rosenthal et al. evaluated 263 patients treated with RFA for osteoid osteomas and reported a clinical success rate of 91%, with only two complications directly related to the procedure [24]. Donkol and colleagues similarly observed a 91% efficacy rate in pediatric patients treated with a single session of RFA, confirming its effectiveness in this population [25].

MWA has also shown promising outcomes. Rinzler et al. documented a 100% success rate employing MWA in 24 pediatric patients with OO [4]. However, in a retrospective study, Reis and colleagues observed an 83% clinical success rate with RFA and a 100% success rate with MWA. They found no notable variance in complications or recurrence between the two treatment modalities after more than 12 months [14]. The current study revealed a clinical success rate of approximately 96%, alongside a recurrence rate as low as 2.8%. These results further reinforce the efficacy of MWA in managing OO, with outcomes that are competitive with those reported for RFA. The low recurrence rate and high clinical success observed in this study suggest that MWA could serve as a reliable treatment option, particularly in cases where traditional RFA might pose challenges or limitations.

In cases of OO treated with MWA, potential complications include skin burns, sensory nerve injuries, and superficial infections at the probe insertion site [3]. Parisot et al. reported three minor complications, including a mild, reversible superficial radial nerve injury with a grade 2 skin burn in one patient and moderate skin burns in two others [3]. On the other hand, Özbalci et al. observed no skin burn complications in their series, with the only significant complication being osteomyelitis in a 2-year-old patient [5]. In this study, 16 complications were recorded, including 13 minor and 3 major complications. The most frequent minor complications were grade 2 skin burns and localized skin numbness, each occurring in 4 cases (2.80%). Regarding the major complications, grade 3 skin burns were seen in 2 cases, and osteomyelitis was reported in 1 case.

Further studies comparing long-term outcomes between these modalities are warranted to solidify MWA’s role in the treatment landscape for OO. The limitation of this review is the heterogeneity among the included studies, which may introduce biases in the assessment of the safety and efficacy of MWA for treating osteoid osteoma. Additionally, the lack of randomized controlled trials limits the ability to draw definitive conclusions regarding the comparative effectiveness of MWA versus other treatment modalities. The review highlights the need for more rigorous prospective studies, including direct comparisons between MWA and RFA, to better understand the relative safety and efficacy of these treatments.

## 5. Conclusion

Percutaneous MWA may represent an efficient choice for the minimally invasive management of OO, demonstrating a minimal risk of complications and recurrence.
